# Supplementary material for: The pace of hospital life: A mixed methods study
Source: PLoS One. 2021 Aug 18;16(8):e0255775. doi: 10.1371/journal.pone.0255775 (PMC8372908; doi:10.1371/journal.pone.0255775)
Supplement: S1 Appendix — (DOCX) [file pone.0255775.s001.docx]

## S1 Appendix

Table 1. Characteristics of survey respondents (*N*=415)

|  |  | ***n*** | ***%*** |
| --- | --- | --- | --- |
| Sex | Male | 84 | 20.9 |
|  | Female | 317 | 78.9 |
|  | Other | 1 | 0.2 |
| Age | 18-24 years | 12 | 2.9 |
|  | 25-34 years | 97 | 23.7 |
|  | 35-44 years | 97 | 23.7 |
|  | 45-54 years | 106 | 25.9 |
|  | > 55 years | 97 | 23.7 |
| Years at hospital | < 1 year | 44 | 10.9 |
|  | 1-2 years | 51 | 12.7 |
|  | 3-5 years | 89 | 22.2 |
|  | 6-10 years | 91 | 22.7 |
|  | > 11 years | 126 | 31.4 |
| Role | Administration/Clerical | 66 | 16.2 |
|  | Allied health professional | 57 | 14.0 |
|  | Management | 33 | 8.1 |
|  | Physician/Medical officer | 74 | 18.1 |
|  | Registered or enrolled nurse | 130 | 31.9 |
|  | Other | 48 | 11.8 |

*Note:* Responses may not equal 415 responses due to missing data
